# DWFed: A statistical- heterogeneity-based dynamic weighted model aggregation algorithm for federated learning

**DOI:** 10.3389/fnbot.2022.1041553

**Published:** 2022-11-24

**Authors:** Aiguo Chen, Yang Fu, Lingfu Wang, Guiduo Duan

**Affiliations:** ^1^School of Computer Science and Engineering, University of Electronic Science and Technology of China, Chengdu, China; ^2^School of Information and Software Engineering, University of Electronic Science and Technology of China, Chengdu, China

**Keywords:** federated learning, statistical heterogeneity, non-IID data, model aggregation algorithm, earth mover's distance

## Abstract

Federated Learning is a distributed machine learning framework that aims to train a global shared model while keeping their data locally, and previous researches have empirically proven the ideal performance of federated learning methods. However, recent researches found the challenge of statistical heterogeneity caused by the non-independent and identically distributed (non-IID), which leads to a significant decline in the performance of federated learning because of the model divergence caused by non-IID data. This statistical heterogeneity is dramatically restricts the application of federated learning and has become one of the critical challenges in federated learning. In this paper, a dynamic weighted model aggregation algorithm based on statistical heterogeneity for federated learning called DWFed is proposed, in which the index of statistical heterogeneity is firstly quantitatively defined through derivation. Then the index is used to calculate the weights of each local model for aggregating federated model, which is to constrain the model divergence caused by non-IID data. Multiple experiments on public benchmark data set reveal the improvements in performance and robustness of the federated models in heterogeneous settings.

## 1. Introduction

As the function of mobile devices, wearable devices, and IoT devices has become more diverse and complex than ever, a tremendous amount of valuable data is generated all the time locally, and huge potential information can be mined through a well-trained statistical model. However, traditional centralized model training requires collecting data in a central node to extract features, which consumes a large amount of time for data transmission and model training because of the tremendous data across the devices. Additionally, it could also cause privacy leakage of sensitive data during transmission. Therefore, federated learning (Konečnỳ et al., 2015; McMahan and Daniel Ramage, 2017; McMahan et al., 2017), a distributed machine learning framework that involves a central server and multiple remote devices, is proposed to address the challenges that centralized methods are confronted with. It enables remote devices to train statistical models locally and only share the parameters of the model to a central server for the aggregation of the federated model, thus providing faster construction of the federated model and privacy of data. Due to these advancements of federated learning, it has been continuously improved and applied in many fields, including smart healthcare (Shamshirband et al., 2021; Rahman et al., 2022; Samuel et al., 2022), industrial internet of things (Sun et al., 2020; Yang et al., 2022), etc. However, federated learning is still confronted with the challenges of model transmission cost and statistical heterogeneity. Specifically, as the parameters of the statistical model are always with high dimensions, frequent parameter uploading can consume lots of transmission time, leading to the low efficiency of federated model training. Besides, statistical heterogeneity results from the non-IID data generated by different devices, which holds various features or labels probability distribution, is proven to have a negative impact on model convergence and accuracy compared with IID data.

To address these challenges, current researchers have proposed several optimization algorithms based on federated learning. Specifically, federated averaging (FedAvg) (McMahan et al., 2017) is such a typical algorithm, which deploys several rounds of local stochastic gradient descent (SGD) on each device and then uploads the parameters of the model to a central server for the model averaging. Several experiments on public benchmark image classification data set (MNIST LeCun et al., 1998, CIFAR-10 Krizhevsky, 2009) and language data set (Shakespeare, 2007) have demonstrated the robustness of FedAvg to train convolutional neural networks (CNN) and long short-term memory (LSTM). However, recent research has found that the statistical heterogeneity caused by non-IID data will increase the model divergence, representing the difference between federated and centralized models, leading to significant accuracy reduction and unstable convergence of federated model.

The research of federated learning dealing with non-IID data mainly focuses on the non-IID label distribution of the data across the clients. To improve the performance of the federated model confronted with non-IID data, Zhao et al. (2018) proposed a data-sharing-based method, which significantly improves the performance of federated average dealing with non-IID data by sharing a small amount of data. In addition, the relation between statistical heterogeneity and earth's mover distance (EMD) is found in their research, which indicates EMD could be an ideal index of statistical heterogeneity. This discovery motivated us to propose DWFed, a dynamic weighted model aggregation algorithm based on a federated averaging algorithm, which quantifies the index of statistical heterogeneity based on EMD, and dynamically computes the weights of model averaging based on the index to minimize the model divergence during federated model training. The most significant difference between FedAvg and DWFed, which is also the main contribution of this paper, is the weights given to the models uploaded by each device. In FedAvg, the weights are simply calculated by the ratio of the data on each device to the total amount of data. The averaging model can represent global optimization objects in IID settings. However, the performance of FedAvg can incredibly shrink as data becomes non-IID because non-IID data makes the weighted sum of local optimization object no longer an unbiased estimation of global optimization object. To overcome the drawback of FedAvg, DWFed calculated weights based on the indexes of statistical heterogeneity called ISH that we quantitatively define through derivation for the first time and is calculated by the EMD between local label distribution and global label distribution. DWFed can well resist the negative impact of non-IID data, and it brings little computation burden to each device as the calculation of weights is simple. However, as each client needs the global sharing label distribution information to calculate its own EMD, respectively, DWFed can better perform in the scenarios where the label information of data is not sensitive, such as hospitals, public driving locations and so on. The detailed introduction of DWFed will be illustrated in Section 3. In addition, experiments on multiple benchmark data sets reveal the improvement of performance and robustness on federated models trained with non-IID data compared with FedAvg. The main contributions of our work are summarized as follows:

1. We quantitatively studied the impact of statistical heterogeneity on federated learning through derivation for the first time.
2. We proposed an index of statistical heterogeneity called ISH, which would decrease as statistical heterogeneity increases.
3. We design a method to dynamically compute model averaging weights by using the index of statistical heterogeneity, which can effectively constrain the model divergence during federated model training.

The rest of our paper is organized as follows. In Section 2, the background and related work of federated learning and the corresponding optimization method is illustrated. The principle of DWFed and its derivation is demonstrated in Section 3. Experiments and evaluations are illustrated in Section 4. Finally, the conclusion of our work is given in Section 5.

## 2. Related work

The notion of federated learning was first introduced in McMahan and Daniel Ramage (2017), and its baseline algorithm is federated stochastic gradient descent (FedSGD), which enables each device to execute one round of SGD locally and upload the model to a central server for weighted model averaging. Then central server distributes the aggregated model to each device for the next round of local SGD, and the whole procedure stops until certain termination conditions are met. Although FedSGD solved the challenges of data transmission and privacy leakage of sensitive data (Bharati and Podder, 2022; Bharati et al., 2022), frequent model uploading and distribution have greatly constrained the performance of federated learning, including slow convergence and low accuracy, and results in the problem of efficiency.

To address the existing challenges, lots of constructive work has been done. In terms of the efficiency of federated learning, Wang et al. (2019) introduced adaptive federated learning that can dynamically compute communication steps with the central server (the rounds of local SGD) in resource-constrained edge computing systems. Faster convergence can be achieved compared with methods where the communication step is fixed. Also, starting from the communication cost, Konečnỳ et al. (2016) greatly reduces the communication cost by utilizing model compression, which decreases the size of the uploading model. Similarly, Sattler et al. (2019) proposed a compression framework called sparse ternary compression (STC), which extends the existing compression technique by enabling downstream compression as well as internalization and optimal Golomb encoding of the weight updates. Additionally, Asad et al. (2020) introduces an algorithm combined with model compression and parameter encryption, which effectively reduces communication overhead while protecting model security. Except for directly reducing communication costs, the efficiency of federated learning could also be improved by resource optimization. For example, Nishio et al. (2013), Sardellitti et al. (2015), and Yu et al. (2016) minimize the computation time and resources consumption based on the joint optimization of heterogeneous data, computation, and communication resources. In contrast, Nishio and Yonetani (2019) maximizes the efficiency of federated model training through client selection based on resources, network conditions, and computation capability, and experiments have proved the enhancement of efficiency.

In terms of robustness in non-IID data, plenty of solutions have been proposed by existing researchers, and we summarize the current federal learning scheme for data heterogeneity in Table 1. For example, Konečnỳ et al. (2015) proposed an optimization algorithm called DSVRG in order to promote the performance of federated learning in non-IID scenarios, in which the distributed optimization algorithm DANE (Shamir et al., 2014) is modified by utilizing SVRG (Johnson and Zhang, 2013) as a local solver to produce an approximate solution for the subproblem of DANE. In addition, some important modifications are taken to improve robustness in federated scenarios, such as flexible local update stepsize and applying the diagonal matrix to adjust the update stochastic gradient value of model. The experiments revealed that DSVRG not only accelerates the convergence but also decreases the test error ratio of federated learning. In 2017, an improved algorithm based on FedSGD called FedAvg (McMahan et al., 2017) is proposed. FedAvg allows devices to synchronously execute several epochs of SGD before uploading the model to a central server for model aggregation, and the convergence of FedAvg is theoretically proved in Li et al. (2019). Experiments on public benchmark data sets also demonstrate that FedAvg has the ideal convergence speed and robustness of training different deep learning models. However, Zhao et al. (2018) found that the performance of FedAvg gradually shrinks as statistical heterogeneity increases. In addition, mathematical analysis is utilized, and the relation between the earth's mover distance of each device and model divergence caused by heterogeneity is discovered. Therefore, a strategy that eases model divergence by sharing a small part of data from the central server to each client is proposed, and experiments have shown that the more data the central server shares, the lower EMD becomes, and the higher accuracy can be obtained. However, the specific mathematical relation between EMD and statistical heterogeneity is not further studied. Chen et al. (2022) proposed an adaptive client selection algorithm ACSFed based on EMD. This algorithm can dynamically calculate the possibility of clients being selected according to the local statistical heterogeneity and previous training performance. Similar to literature (Zhao et al., 2018), an adaptive enhancement method based on data sharing is also proposed in Huang et al. (2018), which improves the efficiency of federated learning. However, data sharing increases the communication burden and raises the risk of privacy leakage. It also breaks the core of federated learning that data should be stored locally instead of sharing. Therefore, recent research has begun to study approaches that can obtain better performance than FedAvg while keeping data locally. For example, Yeganeh et al. (2020) proposed a novel adaptive weighting approach for clients based on meta-information and the comparison with the baseline FedAvg algorithm proves the effectiveness of the scheme. Li et al. (2018) proposed a framework called FedProx, which changes the optimization object by adding the model divergence to the loss function. Experiments prove it can effectively stabilize the training convergence of the federated model because it constrains the difference between the central and local models. Moreover, a creative approach called federated augmentation, which makes data distribution IID on each device by enabling devices to train generative models together to augment data, is proposed in Jeong et al. (2018), and it obtains 95−98% accuracy on MNIST. Xu et al. (2022) proposed a federated learning framework FedLA, which reduces aggregation frequency to improve robustness in heterogeneity scenarios. Furthermore, the cross device momentum (CDM) is implemented to improve the upper limit performance fo the global model. Besides, there is also the idea of dealing non-IID data by combining reinforcement learning with federated learning. For example, Wang et al. (2020) proposes Favor, an experience-driven control framework that intelligently chooses the client devices to participate in each round of federated learning to counterbalance the bias introduced by non-IID data and to speed up convergence. Similarly, Pang et al. (2020) proposed an RL-based intelligent central server with the capability of recognizing heterogeneity, which can help lead the trend toward better performance for most of clients. In 2019, knowledge distillation was applied in federated learning in Li and Wang (2019), which enables each device to train a local model with two parts of data, including private data and public shared data. The outputs of public data are utilized as consensus to adjust each local model, and experiments have shown that the performance of FedAvg can be improved by implementing knowledge distillation. Additionally, there are methods that utilize multi-task learning in federated learning, which are called federated multi-task learning. In federated multi-task learning framework, the learning problem of each client on the local data set is regarded as a separate task rather than a shard of a partitioned data set. MOCHA (Smith et al., 2017) is a typical multi-task federated learning algorithm, which directly solves the challenges of communication efficiency, scatters and fault tolerance. On the basis of MOCHA, Li et al. (2021) proposed a lite framework called Ditto, which simplifies the solver of local subtask by restraining the divergence between local model and global model. Although Ditto's idea of restraining divergence between local model and global model is similar to FedProx, it is essentially different from FedProx, as it not only learns a global model but also learns local, personalized models while FedProx only learns a global model. Experiments on public benchmark dataset reveal that Ditto can enable higher accuracy and stronger robustness relative to state-of-the-art federated learning method. However, as multi-task learning enables each node to train a personalized model locally, a stateful node is also required. This makes this type of technology more challenging to apply in cross-device scenarios. To sum up, there are problems of higher computing and communication burden, privacy leakage, and difficulty in practical application in current research. Therefore, an improved federated learning method that can suppress or solve the above problems while retaining performance must be studied.

**Table 1:** Federated learning for data heterogeneity.

**References**	**Method**	**Dataset**
Konečnỳ et al. (2015)	Distributed optimization	public posts on a large social network
McMahan et al. (2017)	FederatedAveraging	MNIST, CIFAR-10, Shakespeare
Zhao et al. (2018)	Data sharing based on EMD	MNIST, CIFAR-10,KWS
Chen et al. (2022)	Client selection based on EMD	MNIST, Fashion MNIST, CIFAR-10
Huang et al. (2018)	Adaptive enhancement based on data sharing	MIMIC-III, eICU Collaborative research
Yeganeh et al. (2020)	Adaptive weighting based on meta-information	CIFAR-10, Fashion MNIST, HAM10K
Li et al. (2018)	Model divergence	MNIST, FEMNIST, Shakespeare, Sent140
Jeong et al. (2018)	Federated augmentation	MNIST
Xu et al. (2022)	Reduce aggregation frequency based on weight divergence	MNIST, EMNIST, CIFAR-10
Wang et al. (2020)	Client selection based on RL	MNIST, Fashion MNIST, CIFAR-10
Pang et al. (2020)	Recognizing heterogeneity based on RL	MNIST, Fashion MNIST, CIFAR-10
Li and Wang (2019)	Knowledge distillation	MNIST, FEMNIST, CIFAR-10, CIFAR-100
Smith et al. (2017)	Multi-task learning	GLEAM, Human Activity Recognition, Vehicle Sensor
Li et al. (2021)	Personalization federated learning	MNIST, Fashion MNIST, FEMNIST

## 3. Method

To promote the performance of federated learning methods in statistical heterogeneity scenarios, we proposed a dynamical weighted model aggregation algorithm for federated learning called DWFed. The core idea of DWFed is to dynamically calculate the weights of model averaging by using the index of statistical heterogeneity *ISH*. In this section, we will first introduce the core of DWFed in detail, which is the derivation of the index of statistical heterogeneity, and then the overall of DWFed will be demonstrated.

### 3.1. Derivation of model divergence

During federated model training, *K* devices from *N* (*K* < < *N*) are randomly selected and then certain epochs of local stochastic gradient descent (SGD) are executed before uploading model to central server for model aggregation. Specifically, the optimization object is to minimize:


(1) $$\begin{array}{l} \underset{\omega }{\min}f\left(x\right)=\overset{K}{\underset{k=1}{\sum }}\frac{n_{k}}{n}F_{k}\left(\omega \right)whereF_{k}\left(\omega \right)=\frac{1}{n_{k}}\underset{s\in S_{k}}{\sum }f_{k}\left(\omega \right) \end{array}$$

Where *S*_*k*_ is the set of indexs of data points on client k, *n*_*k*_ = |*S*_*k*_| is the data available on device *k*, and $n=\underset{k}{\sum }n_{k}$ is the total data points across the network, *f*_*k*_(ω) refers to the value of loss function of the data on device *k* under the model ω. The procedure of typical federated learning method with *K* selected devices, batch size *b* and learning rate η enables device *k* to iterate local update ω_*k, t*_−η*g*_*k*_ several times, where *g*_*k*_ = ∇*F*_*k*_(ω_*k, t*_) is the gradient computed by the current model ω_*k, t*_ on device *k*, and ω_*k, t*_ = ω_*t*_ when the local update begins. After *K* devices finishing local update and uploading model ω_*k, t*+1_ to central server, model aggregation $\omega_{t+1}=\overset{K}{\underset{k=1}{\sum }}\frac{n_{k}}{n}\omega_{k,t+1}$ is executed on central server, which can also be rewritten as $\omega_{t+1}=\omega_{t}-\eta \overset{K}{\underset{k=1}{\sum }}\frac{n_{k}}{n}\nabla F_{k}\left(\omega_{k,t}\right)$.

In IID settings where training data is uniformly and randomly distributed to each device, the expectation of *F*_*k*_(ω) is equal to *f*(ω), which can be denoted as *E*(*F*_*k*_(ω)) = *f*(*k*), and thus *E*(*g*_*k*_) = ∇*f*(ω). Therefore, the optimal solution can be obtained by updating the model along the descent direction of the gradient and the federated model generated by averaging local models is nearly equal to the centralized model. However, *F*_*k*_(ω) could be an arbitrary approximation to *f*(ω) in non-IID settings, leading to the deviation between federated model and centralized-trained model, which is called model divergence and it can be represented as:


(2) $$\begin{array}{l} \left||\omega^{f}-\omega^{c}||/||\omega^{c}|\right| \end{array}$$

Where ω^*f*^ is the model in distributed settings using federated learning method, and ω^*c*^ is the centralized-trained model. The more significant statistical heterogeneity is, the larger the model divergence is, and the performance of FedAvg can extremely shrink. Therefore, a numerical index of statistical heterogeneity is urgently needed to precisely reflect its influence on the performance of federated learning methods.

### 3.2. Derivation of statistical heterogeneity influence

Through the above derivation and analysis, it can be concluded that the model divergence caused by non-IID data is the main reason leading to decreasing performance of federated learning methods in statistical heterogeneity scenarios. Therefore, we propose a dynamic weighted federated averaging algorithm (DWFed) based on FedAvg which quantitatively defines the index of statistical heterogeneity for the first time and dynamically computes the corresponding weights of model averaging to constrain model divergence. The core idea of DWFed is to calculate comprehensive weights based on the statistical heterogeneity of each selected device and hyperparameters such as learning rate, batch size, and the number of selected devices that are able to make a federated model close to the centralized model and thus constrain the model divergence. Specifically, the centralized model update using SGD can be written as:


(3) $$\begin{array}{l} \omega_{t+1}^{c}=\omega_{t}^{c}-\eta \overset{C}{\underset{i=1}{\sum }}P\left(y=i\right)\nabla F\left(\omega_{t}^{c},x_{y=i}\right) \end{array}$$

In the above equation, $\omega_{t+1}^{c}$ and $\omega_{t}^{c}$ are the weights after *t*+1-th update and *t*-th update respectively, η is the learning rate, *P* is the data distribution which is also the population distribution, and *C* denotes the total classes that data belongs to. In addition, $\nabla F\left(\omega_{t}^{c},x_{y=i}\right)$ denotes the gradients on the data whose class is *i* under current model $\omega_{t}^{c}$. Similarly, we can rewrite the federated model update using FedSGD:


(4) $$\begin{array}{l} \omega_{t+1}^{f}=\omega_{t}^{f}-\eta \overset{K}{\underset{k=1}{\sum }}\overset{C}{\underset{i=1}{\sum }}p_{k}\left(y=i\right)\nabla F_{k}\left(\omega_{k,t}^{f},x_{y=i}\right) \end{array}$$

Where *p*_*k*_ denotes the data distribution on device *k*, and $\nabla F_{k}\left(\omega_{k,t}^{f},x_{y=i}\right)$ is the gradients on data which belongs to class *i* under current local model of device *k*. The superscript of weight ω denotes different settings, that is *c* denotes centralized setting and *f* denotes federated learning setting. To more intuitively compare the model update in two settings, we replace the centralized scenarios with multiple devices with the same data distribution as population distribution, and the number of devices is equal to the number of selected devices in distributed scenarios. The model update in such scenarios is the same as that in centralized scenarios because each device has the same data distribution as population distribution, and the model update can be expressed as:


(5) $$\begin{array}{l} \omega_{t+1}^{c}=\omega_{t}^{c}-\eta \overset{K}{\underset{k=1}{\sum }}\overset{C}{\underset{i=1}{\sum }}P\left(y=i\right)\nabla F_{k}\left(\omega_{k,t}^{c},x_{y=i}\right) \end{array}$$

Therefore, the difference between the federated model and the centralized model, which is inside the numerator part of model divergence, can be rewritten as:


(6) $$\begin{array}{l} \begin{array}{l} \begin{array}{c} \omega_{t+1}^{f}-\omega_{t+1}^{c}=\Delta \omega_{t}+ \end{array} \end{array}\\ \eta \overset{k=1}{\underset{K}{\sum^{\text{​}}}}\overset{i=1}{\underset{C}{\sum^{\text{​}}}}P(y=i)\nabla F_{k}(\omega_{k,t}^{c},x_{y=i})-p_{k}(y=i)\nabla F_{k}(\omega_{k,t}^{f},x_{y=i}) \end{array}$$

where $\Delta \omega_{t}=\omega_{t+1}^{f}-\omega_{t+1}^{c}.$ The above equation illustrates the instability in convergence and low performance of federated learning methods when the statistical heterogeneity leads to uncertain distribution across the devices and thus model divergence increases. To evaluate the model divergence caused by statistical heterogeneity across the devices, EMD can be applied. EMD is a method of calculating divergence by computing the distance between two distributions and Zhao et al. (2018) found model divergence caused by non-IID data can be evaluated with the EMD between the data distribution across devices and population distribution but specific quantitative relation is not given. As EMD denotes the distance between two probability distributions, it can be expressed as the following equation:


(7) $$\begin{array}{l} D_{k}=EMD\left(p_{k},P\right)=||\overset{C}{\underset{i=1}{\sum }}p_{k}\left(y=i\right)-P\left(y=i\right)|| \end{array}$$

A potential problem of the EMD metric is that this metric is not invariant with respect to the automorphism. When the comparison of distributions with various number of labels and the order of labels are different, the EMD will be different. In our method, we quantified the weight divergence by the EMD between the distribution over classes on each device and the population distribution, the data labels across devices are the subset of the global data labels. Thus, the EMD between the data distribution across devices and population distribution is invariant with label alignment. Even if we need to compute EMD of different clients' data distribution, we can also predefine a label order on the central server to obtain the invariant EMD metric. Through this simple method, the EMD between the clients' data distribution and population distribution is a constant. So, we don't have to consider penalization of invariance across different environments. With Equation (7), we can further obtain the index of statistical heterogeneity by introducing EMD into the next stage of derivation. Furthermore, we also propose a dynamic weight aggregation algorithm to compute the corresponding weights of model averaging to constrain model divergence.

### 3.3. ISH and weighted averaging

To address the influence of statistical heterogeneity, we respectively multiply the model of each device with an index called *ISH* which reflects their local statistical heterogeneity, and the model update in distributed settings can be rewritten as:


(8) $$\begin{array}{l} \omega_{t+1}^{f}=\omega_{t}^{f}-\eta \overset{K}{\underset{k=1}{\sum }}ISH_{k}*\nabla F_{k}\left(\omega_{k,t}^{f}\right) \end{array}$$

Since $\omega_{t+1}^{c}$ is determined using SGD as population distribution is known, the optimizing object to minimize model divergence can be expressed as:


(9) $$\begin{array}{l} \min||\eta \overset{K}{\underset{k=1}{\sum }}\left(\nabla F_{k}\left(\overset{c}{\underset{k,t}{\omega }}\right)-ISH_{k}*\nabla F_{k}\left(\overset{f}{\underset{k,t}{\omega }}\right)+\frac{1}{K*\eta }\Delta \omega_{t}\right)|| \end{array}$$

Based on the idea of greedy algorithm, we can optimize (Equation 9) by minimizing each part of it, which is:


(10) $$\begin{array}{l} ||\nabla F_{k}\left(\omega_{k,t}^{c}\right)-ISH_{k}*\nabla F_{k}\left(\omega_{k,t}^{f}\right)+\frac{1}{K*\eta }\Delta \omega_{t}|{|}_{k=1,2,...,K}\to 0 \end{array}$$

Therefore, the index of statistical heterogeneity in device *k* can be calculated by the following formula:


(11) $$\begin{array}{l} ISH_{k}=\frac{\left||\nabla F_{k}\left(\omega_{k,t}^{c}\right)+\frac{1}{K*\eta }\Delta \omega_{t}|\right|}{\left||\nabla F_{k}\left(\omega_{k,t}^{f}\right)|\right|} \end{array}$$

Based on formula (6) and (7), the index *ISH*_*k*_ can be further calculated as:


(12) $$\begin{array}{l} ISH_{k}=\frac{1-\frac{1}{K}D_{k}}{1+D_{k}} \end{array}$$

After the *ISH* of each selected device *k* is obtained, they are respectively transmitted to the central server along with the local model by each device. Then the weights of each local model are calculated by executing the normalization of indexes to make sure the sum of weights is equal to 1:


(13) $$\begin{array}{l} \alpha_{k}\leftarrow ISH_{k}/\overset{K}{\underset{k=1}{\sum }}ISH_{k} \end{array}$$

Finally, the central server executes weighted model aggregation following formula (8), and returns the aggregated model to each selected device for a new round of federated local model training, which is also the end of a communication round.

### 3.4. Algorithm implementation

After deriving the statistical heterogeneity index *ISH*, we will describe our DWFed algorithm in detail.

The DWFed algorithm conducted by multiple rounds of communication among central server and clients. A complete communication round includes local data training, aggregation weight calculation, model and weight transmission, model aggregation, and model distribution. The complete pseudo-code of DWFed is given in Algorithm 1. At the beginning of the DWFed, the central server first initializes the weights and distributes the weights to a randomly selected set of clients. After receiving the weights, the clients first calculate *ISH* according to formula (12), then each clients execute one round of SGD locally, and finally clients transmit the updated weights and *ISH*_*k*_ to the central server. The central server calculates the aggregation weights α_*k*_ based on the parameters uploaded by the client and completes the model aggregation. This is the whole process of a round of communication, and the algorithm keeps repeating until prescribed communication rounds are met.

**Algorithm 1:**
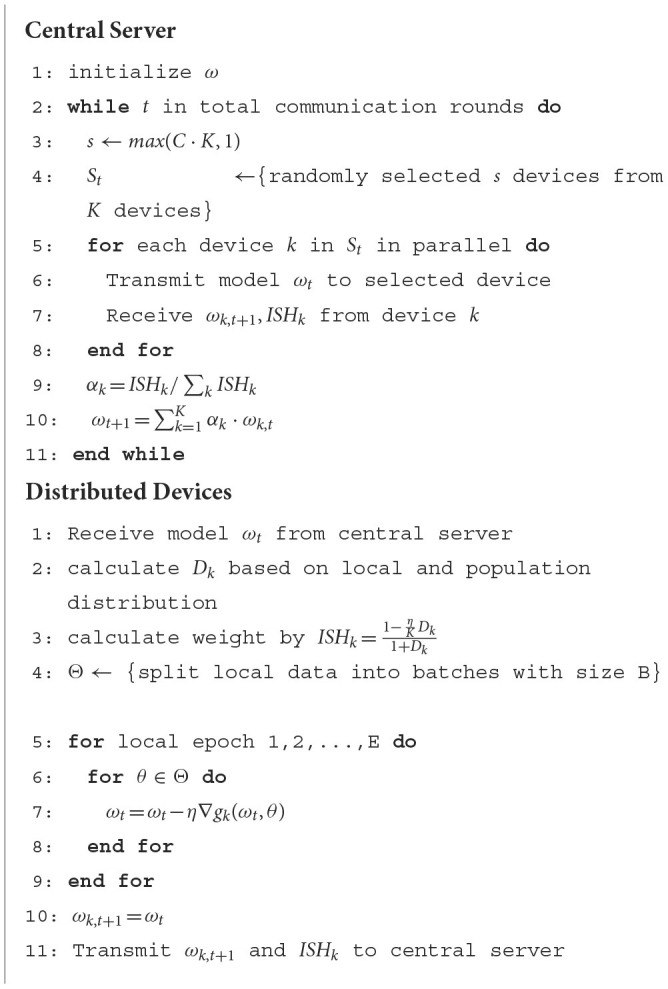
Dynamic weighted federated averaging. The *K* clients are selected from *N* with the fraction *C* and are indexed by 1, 2, …, *k*; learning rate is expressed as η, *B* and *E* respectively denote the batch size and training epochs used in local stochastic gradient descent. Specifically, *D*_*k*_ denotes the EMD between the data distribution on device *k* and population distribution.

It can be seen from Algorithm 1 that DWFed only adds little computational and communication load. The process of calculating *ISH* on the clients is simple and straightforward. Furthermore, clients only need to upload one additional float value to central server. After the normalization of weights, the central server can aggregate the model, which is also an effective calculation.

Further, to prove and evaluate the performance of our algorithm, multiple comparison experiments are executed, and the details of the experiments will be illustrated in the next section.

## 4. Experiment and evaluation

In this section, the details of comparison experiments and the evaluation of the results are illustrated. We will firstly introduce the methods used to distribute data to each selected device, which can generate different degrees of statistical heterogeneity on each device. Then the experimental environment will be detailly illustrated, including the total number of devices, the selection fraction, and the model implemented on each device. Finally, experimental results and evaluation are demonstrated.

### 4.1. Data allocation and experiment setup

In this paper, two types of non-IID data are generated to compare the performance of DWFed and FedAvg in different degrees of statistical heterogeneity, which are two extreme cases of data distribution: (a) 1-class non-IID, where each device only holds data partition from only a single class, and (b) 2-class non-IID, where the sorted data is divided into 20 partitions and each client is randomly assigned 2 partitions from 2 classes.

In terms of devices, we simulate 100 devices in total, and respectively with the fraction value of *C* = 0.1 or 0.2 to randomly select 10 or 20 devices to participate in federated training. As for baseline algorithm, FedAvg with fraction value 0.2 is selected, since it could obtain the best performance in prior experiments. At the beginning of the experiment, training data is generated in the form of 1-class non-IID or 2-class non-IID and distributed to all devices, then the central server randomly selects 10 devices for model update and distributes initialized model to these devices. After the local SGD model update, the selected models upload their locally updated model and the averaging weights to a central server. After the normalization of weights, the central server executes model aggregation by weighted averaging of models. The whole procedure keeps repeated until it reaches the prescriptive communication rounds.

The experiments are all implemented in the same machine with Intel (R) Core(TM) i5-7300HQ, CPU @ 2.50GHz, and 16-Gb RAM. The FedAvg and DWFed are both implemented in Pycharm with Python of 3.6 version, which installed TensorFlow GPU version and other useful packets.

### 4.2. Experimental evaluation

In the experiments, three different kinds of data set are used to evaluate the performance of DWFed, which are MNIST, Fashion MNIST (FMNIST), and CIFAR-10, and all of them are data sets for image classification tasks with 10 outputs. Therefore, convolution neural networks (CNNs) are implemented on each device. Specifically, for MNIST and Fashion MNIST training, the structure of CNNs is the same because they have the images with the same size and single image channel, and they both have the same amount of training set and test set. We adopt the same network structure as literature (McMahan et al., 2017). There are two 5 x 5 convolution layers (the first with 32 channels, and the second with 64 channels, each followed with 2 x 2 max pooling), a full-connected layer with 512 units and ReLu activation, and a final softmax output layer. However, CIFAR-10, which contains 10 classes of the three-channel image with size [32, 32], and thus the CNNs for CIFAR-10 training has 9 layers, two more full-connected layers are added compared with CNNs for MNIST and Fashion MNIST. Specifically, there are two 5 x 5 convolution layers (both of them have 64 channels, and followed with 2 x 2 max pooling), three full-connected layers with 768, 384, and 192 units respectively and ReLu activation, and a finall softmax output layer. As for hyperparameters of the CNNs model, the same hyperparameters are set in FedAvg and DWFed. Specifically, we set learning rate η = 0.01, batch size *B* = 10, epochs for every local update *E* = 5, number of communications *ncom* = 100 for MNIST and *ncom* = 1, 000 for Fashion MNIST and CIFAR-10. The aggregated model is validated with corresponding test data in every 20 rounds of communication.

**Figure 1:**
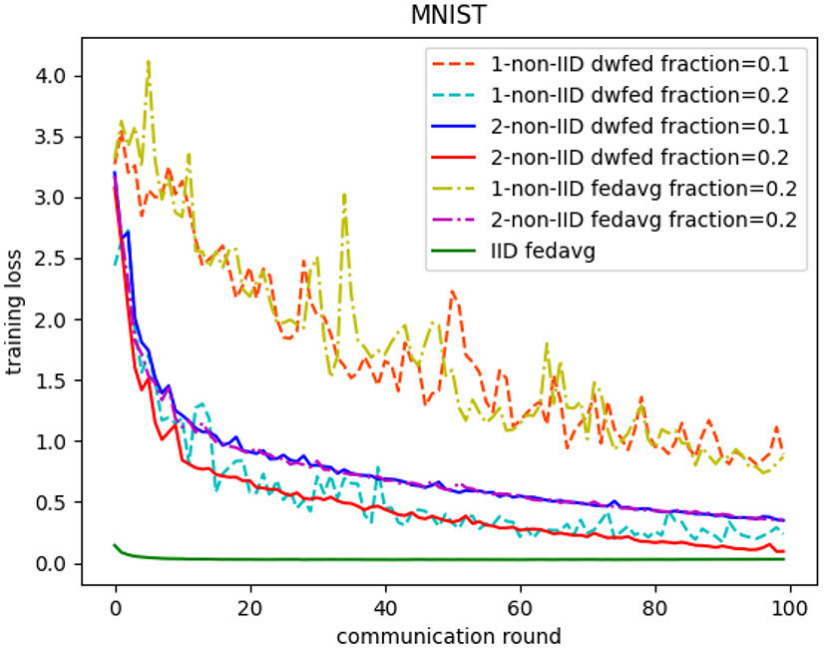
Training loss on MNIST.

**Figure 2:**
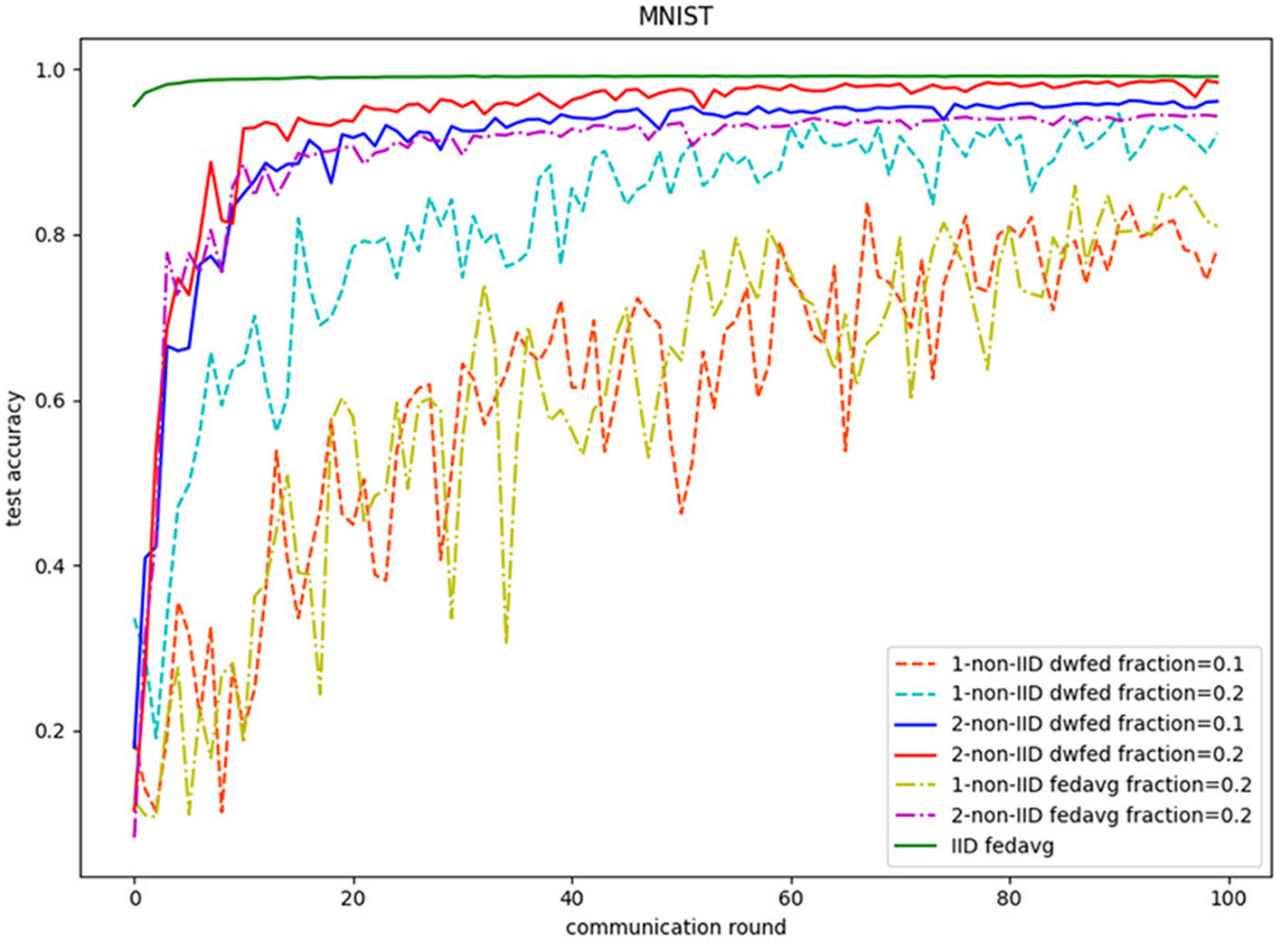
Test accuracy on MNIST.

**Figure 3:**
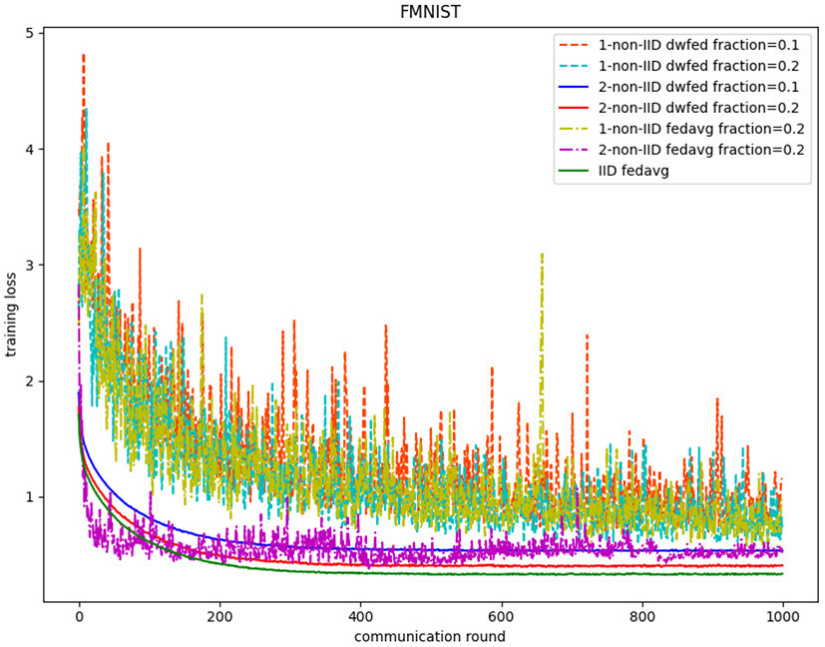
Training loss on fashion MNIST.

**Figure 4:**
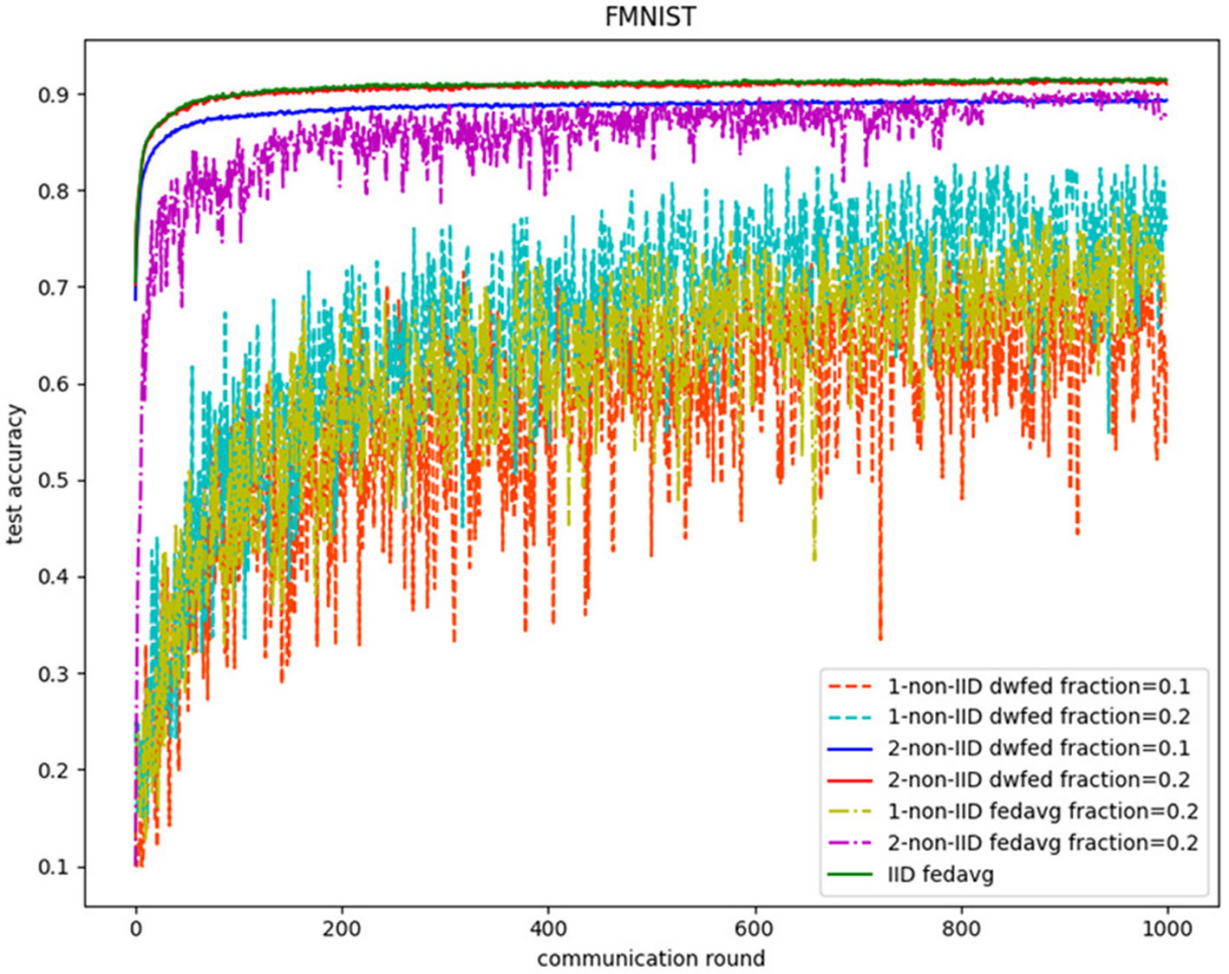
Test accuracy on fashion MNIST.

The evaluation of our algorithm is achieved by comparing the performance of FedAvg and DWFed on three data sets under three scenarios of statistical heterogeneity (1-class non-IID, 2-class non-IID, and IID). The experiment results on MNIST are demonstrated in Figures 1, 2. The Figure 1 reveals the improvement of convergence using DWFed as the curve of DWFed drops faster and fluctuates less than FedAvg both in 1-class and 2-class non-IID scenarios. Moreover, DWFed with *c* = 0.1 is able to have the similar performance as FedAvg with *c* = 0.2, both in 1-class and 2-class non-IID scenarios and DWFed with fraction value 0.2 in 1-class non-IID scenario even have lower training loss than FedAvg in 2-class non-IID scenario, which significantly reveals DWFed has better performance on training convergence than FedAvg. The enhancement of DWFed on the accuracy of MNIST is illustrated in Figure 2, as the accuracy reaches certain level (80% for 1-class non-IID and 60% for 2-class non-iid) faster and obtains higher final accuracy than FedAvg. Moreover, the test accuracy of DWFed with fraction value of 0.2 in 1-class non-IID scenario is very close to FedAvg in 2-class non-IID scenario. Figures 3, 4 reveal the performance of DWFed and FedAvg on Fashion MNIST in three scenarios. As can be concluded from Figure 3, the loss curves of DWFed in two non-IID scenarios generally fluctuate less than FedAvg, and DWFed with less selection fraction can have similar performance to FedAvg with higher fraction. Besides, the curves of DWFed are closer to the curve in the IID scenario than FedAvg both in 1-class and 2-class scenarios, which reveals the improvement in convergence by implementing DWFed.

Figure 4 demonstrates the comparison test accuracy of DWFed and FedAvg on Fashion MINIST. As can be seen from the Figure, the DWFed with selection fraction value 0.2 obtains 90.2% for test accuracy of FMNIST after 1,000 communication rounds in 2-class non-IID scenarios, which is 2.4% more than FedAvg and only 1.3% less than FedAvg in IID scenario. Additionally, DWFed with fraction value of 0.1 obtains even higher accuracy than FedAvg with fraction 0.2 in 2-class non-IID scenario. However, the accuracy of DWFed and FedAvg are both lower than 80% in 1-class non-IID scenario, but DWFed still obtains 77.8% accuracy, which is higher than the 75% accuracy of FedAvg.

**Figure 5:**
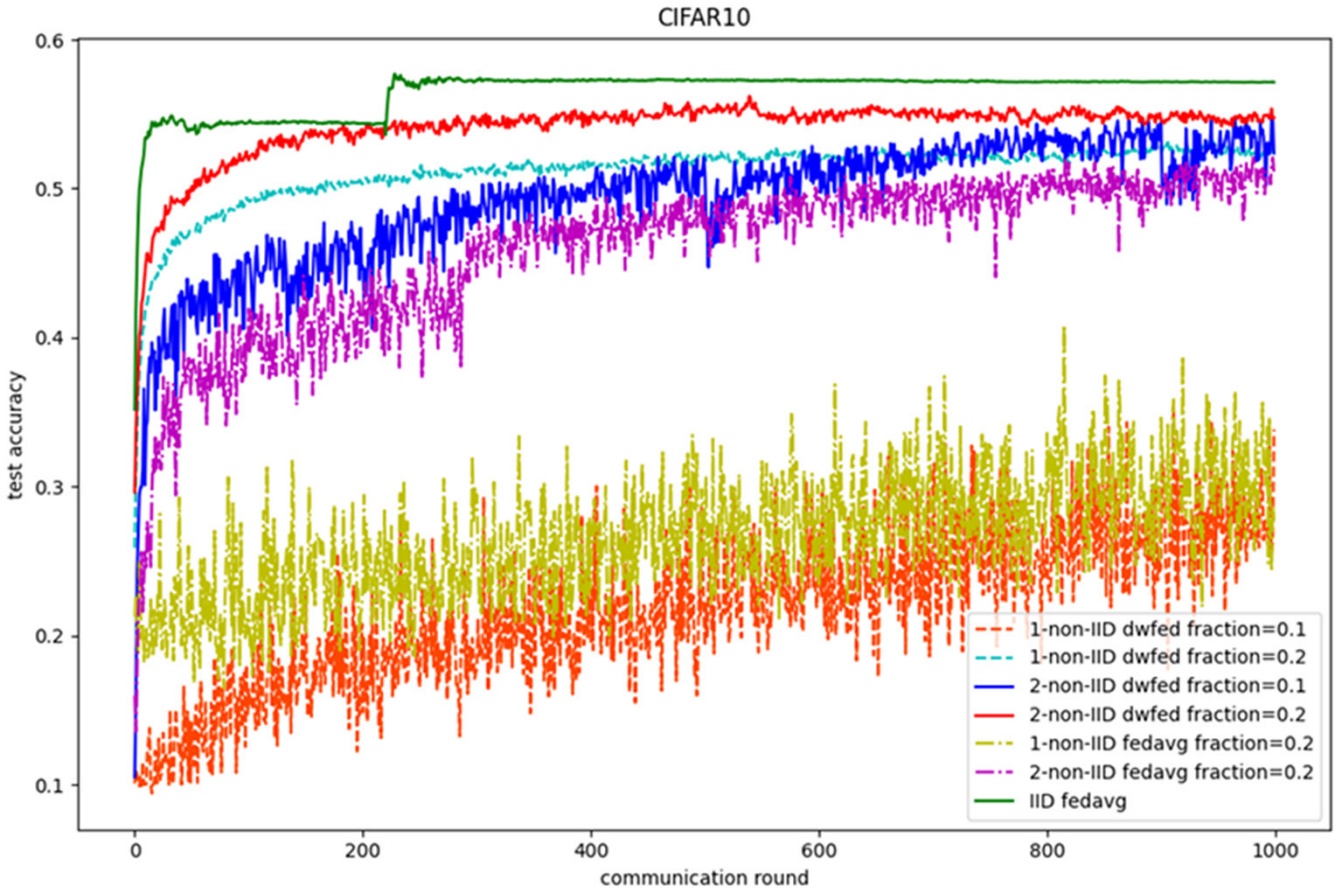
Test accuracy on CIFAR10.

In the terms of CIFAR-10, the performance of federated learning methods on CIFAR-10 can also be significantly improved with the implementation of DWFed. As can be seen in the Figure 5, DWFed with fraction value 0.1 and 0.2 both obtain higher test accuracy than FedAvg and has smaller difference with FedAvg in IID scenario. Moreover, DWFed with fraction value 0.2 in 1-class non-IID scenario even have higher accuracy than FedAvg in 2-class scenario, which reveals the significant improvement of implementing DWFed in the non-IID scenario on CIFAR-10 dataset.

Then, the experimental results of accuracy reduction and convergence speed on three public benchmark data sets are shown in Table 2 (both FedAvg and DWFed have the same experimental settings, such as learning rate and selection fraction value). As can be concluded from Table 2, DWFed has lower accuracy reduction and faster speed of convergence in two non-IID scenarios than FedAvg, which intuitively reflects the superiority of DWFed. In conclusion, DWFed is able to significantly improve the robustness and performance of federated learning methods in non-IID scenarios, as it can reach similar performance to FedAvg with less devices selected each training epoch and higher performance with the same device selection fraction. Specifically, the more stable convergence and higher accuracy are achieved compared with FedAvg.

**Table 2:** Performance of FedAvg and DWFed.

**Data set**	**non-IID**	**FedAvg**	**DWFed**
**Accuracy reduction**
MNIST	1-class	6.16%	4.48%
	2-class	2.40%	0.60%
FMNIST	1-class	15.00%	10.21%
	2-class	3.80%	1.20%
CIFAR-10	1-class	29.39%	21.53%
	2-class	5.54%	1.39%
**Round of convergence**
MNIST	1-class	74	50
	2-class	12	8
FMNIST	1-class	800	620
	2-class	770	560
CIFAR-10	1-class	910	750
	2-class	800	520

**Figure 6:**
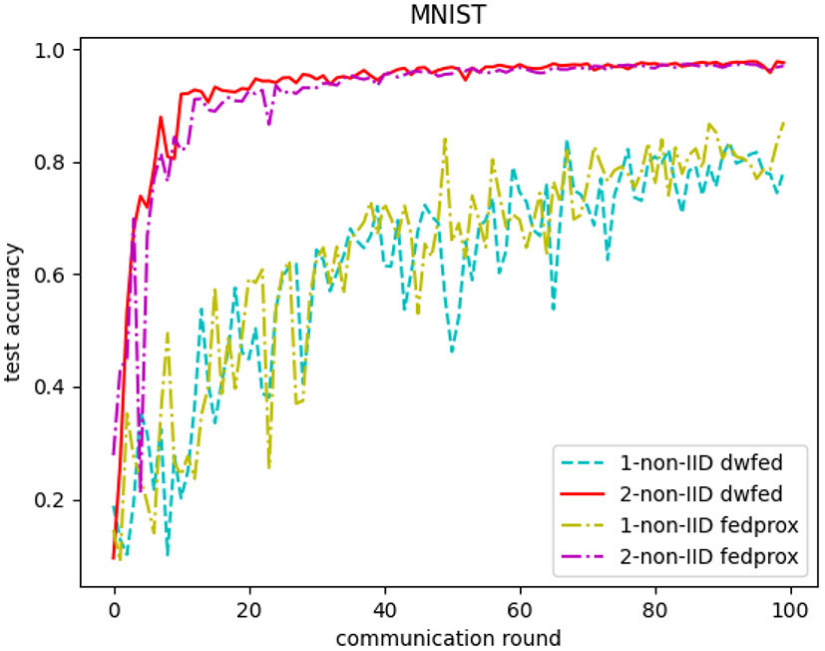
Test accuracy of FedProx and DWFed on MNIST.

**Figure 7:**
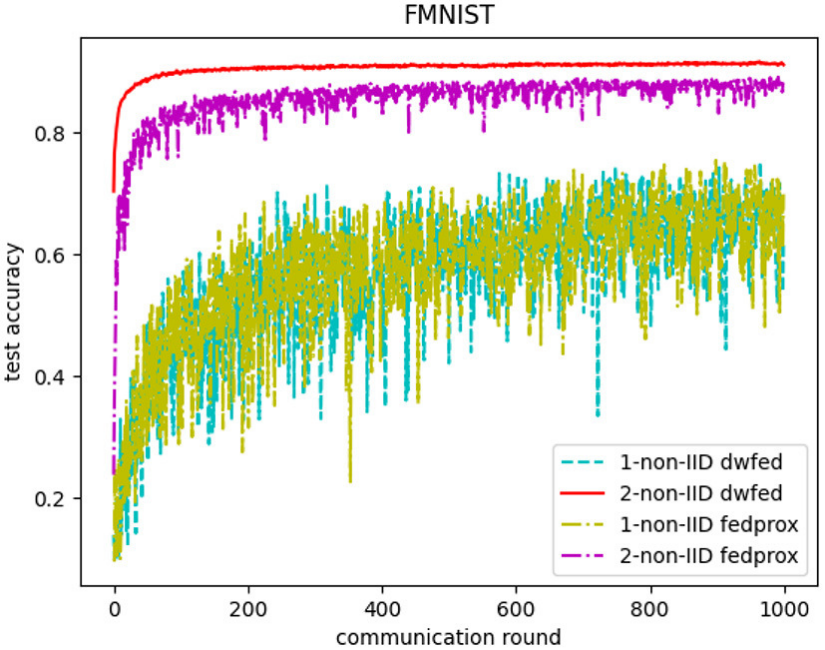
Test accuracy of FedProx and DWFed on FMNIST.

In addition, the comparative experiments of DWFed and FedProx are carried out in MNIST and FMNIST, and the results are shown in Figures 6, 7. As can be concluded from Figures 6, 7, DWFed can obtain better performance than FedProx on MNIST and FMNIST in 2-class non-IID scenario, but FedProx can achieve better results in 1-class non-IID scenario on both datasets. The results above has revealed that DWFed is able to have significant advantages in scenarios with weaker statistical heterogeneity, as it has better performance and lower computational burden. However, DWFed can not achieve better performance than FedProx in scenarios with heavier statistical heterogeneity, because FedProx introdues proximal term to limit the impact of local updates in heterogeneous networks, thus FedProx focuses computation energy to promote performance.

## 5. Conclusion

Federated Learning will play an essential role in future computation mode as the computation capability of remote edge devices enhances and local data privacy increases. However, the statistical heterogeneity can result in model divergence, which significantly influences the performance of federated learning methods, such as shrinking accuracy and unstable convergence. In this paper, we proposed a dynamic weighted model aggregation algorithm for federated learning called DWFed and further quantified the index of statistical heterogeneity using EMD through derivation. Then the model aggregation weights of each device can be calculated by the corresponding index, and the local model divergence can be effectively constrained by multiplying weights in model aggregation. Experiments on three different data set reveal the better performance of DWFed than FedAvg.

Moreover, compared with the SoA methods, such as FedProx, DWFed can obtain better performance in scenarios with weaker statistical heterogeneity, and achieve similar or slightly worse performance in a scenario with heavier statistical heterogeneity. Furthermore, DWFed adds little computational and communication load because the calculation of ISH is straightforward (simple) and only one additional float value is uploaded, in contrast to FedProx, which would use much more computational resources to improve performance. Nevertheless, model divergence can possibly be improved. There is still the challenge of model protection, and additional research is required to find a solution to the problems so that federated learning methods can be applied more effectively in the future.

## Data availability statement

The original contributions presented in the study are included in the article/supplementary material, further inquiries can be directed to the corresponding author.

## Author contributions

AC and YF contributed to conception and design of the study. YF, GD, and LW performed the statistical analysis and wrote the first draft of the manuscript. All authors contributed to manuscript revision and read and approved the submitted version.

## Funding

This work was supported by the National Natural Science Foundation of China (No. U19A2059) and by the Ministry of Science and Technology of Sichuan Province Program (No. 2021YFG0018 & No. 20ZDYF0343).

## Conflict of interest

The authors declare that the research was conducted in the absence of any commercial or financial relationships that could be construed as a potential conflict of interest.

## Publisher's note

All claims expressed in this article are solely those of the authors and do not necessarily represent those of their affiliated organizations, or those of the publisher, the editors and the reviewers. Any product that may be evaluated in this article, or claim that may be made by its manufacturer, is not guaranteed or endorsed by the publisher.
